# ASSOCIATION BETWEEN NEIGHBORHOOD CHARACTERISTICS AND DEMENTIA: AN ECOLOGICAL ANALYSIS

**DOI:** 10.1093/geroni/igz038.3209

**Published:** 2019-11-08

**Authors:** Dana M AlHasan, Matthew Lohman, Maggi Miller, Jana A Hirsch, Bo Cai

**Affiliations:** 1 University of South Carolina, Columbia, South Carolina, United States; 2 Drexel University, Philadelphia, Pennsylvania, United States

## Abstract

Research has examined the relationship between neighborhood environments and cognitive decline, yet few have investigated the role of neighborhood characteristics specifically on dementia. This ecologic study examined the geographic distribution of dementia incidence and investigated ecologic associations between census-tract neighborhood characteristics and diagnosed dementia case incidence from 2010-2014 in the South Carolina (SC) Alzheimer’s Disease Registry. Analyses took place on the census-tract level (n=1089) with population ≥1. Neighborhood measures came from the Decennial Census, American Community Survey, Rural Commuting Area Code, and County Health Rankings. To estimate the ecologic association between neighborhood characteristics and dementia incidence, we conducted a zero-inflated, negative binomial mixed-effects model. The overall age-sex standardized dementia incidence rate was 2,885.4 per 100,000 people per census tract from 2010-2014 in SC (95% CI: 2857-2913.7). In an adjusted model, neighborhood characteristics had a significant association with dementia incidence. Rural and small urban census tracts had 62% (IRR=0.38; 95% CI= 0.27-0.54) and 58% (IRR=0.42; 95% CI= 0.29-0.60) lower dementia incidence, respectively, among ≥50 years old compared to urban census tracts while adjusting for neighborhood median-household income, racial composition, commute time, and age structure. The results from this study show a negative relationship between rural neighborhoods and dementia, contrary to previous findings. However, lower access to care in rural neighborhoods can result in lower detection rates and thus could present a reporting bias. Future research should investigate additional census-tract neighborhood characteristics (e.g. green space, pollution rates or psychosocial stress) that contribute to lower dementia rates in rural areas.

